# Mindfulness based cognitive therapy improves frontal control in bipolar disorder: a pilot EEG study

**DOI:** 10.1186/1471-244X-12-15

**Published:** 2012-02-29

**Authors:** Fleur M Howells, Victoria L Ives-Deliperi, Neil R Horn, Dan J Stein

**Affiliations:** 1 Department of Psychiatry, Faculty of Health Sciences, University of Cape Town, Observatory 7925, South Africa; 2 Department of Human Biology, Faculty of Health Sciences, University of Cape Town, Observatory 7925, South Africa

## Abstract

**Background:**

Cognitive processing in Bipolar Disorder is characterized by a number of attentional abnormalities. Mindfulness Based Cognitive Therapy combines mindfulness meditation, a form of attentional training, along with aspects of cognitive therapy, and may improve attentional dysfunction in bipolar disorder patients.

**Methods:**

12 euthymic BD patients and 9 control participants underwent record of electroencephalography (EEG, band frequency analysis) during resting states (eyes open, eyes closed) and during the completion of a continuous performance task (A-X version, EEG event-related potential (ERP) wave component analysis). The individuals with BD completed an 8-week MBCT intervention and record of EEG was repeated.

**Results:**

(1) Brain activity, individuals with BD showed significantly decreased theta band power, increased beta band power, and decreased theta/beta ratios during the resting state, eyes closed, for frontal and cingulate cortices. Post MBCT intervention improvement over the right frontal cortex was seen in the individuals with BD, as beta band power decreased. (2) Brain activation, individuals with BD showed a significant P300-like wave form over the frontal cortex during the cue. Post MBCT intervention the P300-like waveform was significantly attenuated over the frontal cortex.

**Conclusions:**

Individuals with BD show decreased attentional readiness and activation of non-relevant information processing during attentional processes. These data are the first that show, MBCT in BD improved attentional readiness, and attenuated activation of non-relevant information processing during attentional processes.

## Background

Although significant advances have been made in understanding the psychobiology of bipolar disorder, the specific deficit underlying its symptoms remains unclear. One important possibility is that attentional dysfunction is a key intermediate phenotype for understanding the pathogenesis of bipolar disorder (BD) [1-3]. Thus, attentional dysfunction in BD is a consistent finding in the literature [1,3-6]. Electroencephalographic (EEG) studies have made a particular contribution to this work, reporting asymmetrical activity (at rest) and activation (during task) in bipolar disorder; however these asymmetrical differences are dependent on mood state [4,7,8]. In euthymic BD asymmetry can be seen during high task difficulty and is opposite to that which occurs in controls i.e. completion of difficult anagrams increased left frontal alpha activation while control participants showed decreased alpha activation [9]. Similarly EEG Event-related potential (ERP) studies report a myriad of deficits during attentional tasks during polar mood states, such as reduced P300 amplitude and latency [10,11]. However when euthymic, attentional task P300 amplitude and latency are similar to controls [10]. An earlier ERP auditory attentional component such as the P50, related to auditory sensory gating, is diminished in schizophrenia and has also been reported in bipolar disorder if they present with a florid history of psychosis [12]. At present it still remains unclear which attentional components are involved euthymic BD during attentional processes.

Attentional dysfunction in BD is suggested to result from interfering neural processing that is not related to the task at hand, this superfluous or interfering information processing has been suggested to result from over activity of emotional brain areas [2] and 'weak' regulation of approach systems [13]. To overcome the over activity of emotional brain areas individuals with BD show increased activity of attention related brain areas [2]. This suggests that if we are able to reduce interfering neural processing and/or improve regulation of approach systems we would improve attentional processing in BD. Mindfulness based cognitive therapy (MBCT) combines meditative practices and aspects of cognitive therapy, which is based on the mindfulness-based stress reduction program [14]. The aim of meditative practices is to "reduce or eliminate irrelevant thought processes through training of internalized attention" [15]. The aim of mindfulness mediation is to "facilitate adaptive coping with life stressors and enhance emotional well-being" [16], for a recent review see [17]. The aim of MBCT is to incorporate the above and include cognitive behavioral therapy to "teach skills that will enable a greater awareness of thoughts, without judgment, and viewing negative (positive and neutral) thoughts as passing mental events rather than as facts" [18]. While some research in major depression has focused on EEG variables relevant to mindfulness [19-21], limited research has been conducted in BD with these forms of therapy, and no EEG studies have been conducted to our knowledge [18,22].

The aims of the present study were therefore to (1) determine brain activity and activation differences between euthymic BD and controls, at rest (EEG band frequency analysis) and during an attentional task, a continuous performance task (EEG ERP wave component analysis). (2) To determine the effects of MBCT on brain activity and activation differences in euthymic BD, at rest (EEG band frequency analysis) and during an attentional task, a continuous performance task (EEG ERP wave component analysis).

## Methods

### Participants

Twenty-one participants were recruited for the present study. Twelve individuals with bipolar I in euthymic state (37 ± 7.3 yrs, 10 females and 2 males, manic episodes 2.8 ± 2.6, depressive episodes 2.8 ± 3.0) and 9 control individuals (29 ± 6.4 yrs, 7 females and 2 males) with no prior psychiatric history and no first degree relatives with bipolar diagnoses. The study was approved by the Faculty of Health Sciences Human Ethics Committee of the University of Cape Town, and the participants signed informed consent (UCT HSF HREC 078/2009). The study was conducted in accordance with the Declaration of Helsinki, 2000 [23].

All BD participants were medicated. All BD participants were receiving mood stabilizers, 8 of which were receiving lithium. Other prescribed medications included antipsychotics (8 participants), antidepressant (2 participants), and or anxyolytic (2 participants) medications. Co morbidities present in the BD cohort included: impulse control disorders (1 participant with trichotillomania and with compulsive buying, 1 participant with compulsive buying), anxiety disorders (3 participants with panic disorder and agoraphobia), and substance abuse disorders (1 participant with a past history of alcohol and cannabis dependence, 1 participant with a past history of benzodiazepine and diet pill dependency, 1 participant with current alcohol abuse).

### Experimental design

Each of the participants underwent a structured clinical interview (SCID), to confirm diagnosis in bipolar group, and exclude mood disorders in the control group. Each of the participants underwent continuous electroencephalographic (EEG) record of their brain wave activity, at rest with eyes open for 3 min, with eyes closed for 3 min, and during a continuous performance task of approximately 10 min long. The bipolar participants completed an 8 week MBCT intervention. After the MBCT intervention the participants with bipolar disorder completed the EEG protocol again.

On each day of the EEG study, clinical scales were administered; these included the Young Mania Rating Scale (YMRS) and the Hospital Anxiety and Depression Scale (HADS) in order to confirm mood state. The EEG record was obtained within an hour session, between 09 h00 and 13 h30. The EEG session was completed in a quiet, dimly lit room to reduce distraction. The researcher conducting the experiments was with the participant at all times during the testing. All stages of the testing session were programmed in Eprime 1.1, which sent digital inputs of stimuli to Acqknowledge 4.1 (Biopac Systems Inc.) software, via an MP150 Biopac acquisition system, that also collected the EEG data (EEG 100 C amplifier modules).

### Sustained attentional task

A visual A-X continuous performance task was completed by the participants. Participants were required to respond with button press on presentation of the letter X if preceded by the letter A. All non target stimuli were letters of the alphabet other than A and X and did not include any vowels, to prevent word formation of randomly presented letters. Inter-stimuli intervals were set to 1000 msec, duration of target and non-target stimuli were for 500 msec. Fifty target stimuli were presented during the task.

### Electroencephalography (EEG)

EEG data was recorded with the use of a bioamplifier system produced by Biopac Systems Incorporated (MP150 system with 10 EEG100C amplifiers). The data was stored and processed with Acqknowledge 4.1 software from Biopac Systems Inc. A standard 10/20 linked ears reference montage EEG system was used, and individuals were grounded peripherally. Brain frequency activity and event-related potentials (ERPs) were obtained from: F_3_, F_4_, C_3_, C_4_, P_3_, and P_4_. The data was sampled at 500 Hz and band pass filtered FIR with a Hamming window (0.5 - 30 Hz). Electrooculogram (EOG) electrodes were used to assist in removal of EOG artifact, through an automated independent component analysis within Acqknowledge 4.1.

To obtain relative frequency power the data underwent Fast Fourier transform after channel filtering for resting conditions (eyes open and eyes closed) and the continuous performance task. Absolute power values were obtained and the relative values for theta (θ, 4-7 Hz), alpha (α, 7-14 Hz), and beta (β, 15-30 Hz) frequency bands were reported.

To obtain the ERP waveforms during the sustained attention task, epochs 200 msec prior to stimulus and 800 msec post target presentation were extracted to give a 1 sec window. This was also performed for both the target (letter X) and the cue (letter A). The extraction was set to reject ERPs that were greater than or less than 100 μV. This was followed by visual inspection of the average ERP for each participant. Each ERP was baseline corrected for 200 msec prior to stimulus presentation. The prominent wave components extracted during the target: early positive component (0-100 msec window), negative component (200-400 msec window), and late positive component (300-500 msec window). During the cue, P300-like wave component was extracted (200-400 msec window).

### Mindfulness based cognitive therapy (MBCT)

The MBCT intervention for BD [18] was developed from the 8-week MBCT program for major depression [24]. Specifically, the 8-week MBCT intervention for BD included psycho-education with a specific focus on mania and the early warning signs of mania and depression [18]. During their participation in the present study, participants were encouraged to continue with all of their outpatient appointments, medications and other services they were receiving. None of the participants reported using non-MBCT meditation or yoga for therapeutic purpose during their participation in the present study. The MBCT intervention was led by an experienced therapist with recognized expertise in the delivery of cognitive therapy and MBCT.

### Statistical analysis

Statistica 10 was used for the statistical analyses. Independent-sample *t*-tests were performed between control participants and individuals with bipolar disorder. Dependent-sample *t*-tests were performed between the individuals with bipolar prior and post their mindfulness based intervention. No further analyses were performed due to the pilot nature of these data. Data were reported as mean ± STDEV, significant values *p *< 0.05 and when refer to tendencies *p *< 0.1.

## Results

The individuals with bipolar disorder reported had higher mean YMRS (3.41 ± 3.02) than control individuals (0.00). There was no difference in the Hospital Anxiety (bipolar_anxiety _= 8.41 ± 4.48, control_anxiety _= 7.33 ± 4.58) and Depression (bipolar_depression _= 5.08 ± 2.93, control_depression _= 3.33 ± 2.59) rating scores between the groups. There was no significant change in Hospital Anxiety (bipolar_anxiety _= 7.83 ± 3.51) or Depression (bipolar_depression _= 5.83 ± 4.76) rating scores post MBCT.

### Brain activity (resting states)

Individuals with BD showed decreased theta band power during the resting condition with eyes closed, over the frontal cortex (F_3 _*t *= 2.29; F_4 _*t *= 2.75, *p *< 0.05) and cingulate cortex (C_3 _*t *= 2.19; C_4 _*t *= 2.36, *p *< 0.05) (Table 1). Individuals with bipolar disorder showed increased beta band power during the resting condition with eyes closed, over the frontal cortex (F_3 _*t *= -2.43; F_4 _*t *= -2.76, *p *< 0.05) and cingulate cortex (C_3 _*t *= -2.25; C_4 _*t *= -2.51, *p *< 0.05) (Table 1). No significant difference was seen over the parietal cortex (Table 1). These differences in theta and beta band power over the frontal and cingulate cortex can be expressed as differences in the theta/beta ratios during the resting condition with eyes closed over the frontal (F_3 _*t *= 2.68; F_4 _*t *= 3.42, *p *< 0.05) and cingulate cortex (C_3 _*t *= 2.29; C_4 _*t *= 2.64, *p *< 0.05) (Table 1). No differences were seen over the parietal cortex (Table 1).

**Table 1:** Relative EEG band power and theta/beta ratios during resting conditions (eyes open, eyes closed) and during a continuous performance task

	Control			Bipolar		Bipolar MBCT		Control		Bipolar			Bipolar MBCT		
	
	MEAN	STDEV		MEAN	STDEV	MEAN	STDEV	MEAN	STDEV		MEAN	STDEV		MEAN	STDEV
				**Left Frontal (F_3_)**				**Right Frontal (F_4_)**							

**Resting Eyes Open**															
*relative theta band power (4-7 Hz)*	0.29	*0.10*		0.23	*0.07*	0.27	*0.09*	0.30	*0.09*		0.24	*0.06*		0.27	*0.09*
*relative alpha band power (7-14 Hz)*	0.35	*0.03*		0.38	*0.11*	0.33	*0.06*	0.35	*0.03*		0.35	*0.05*		0.34	*0.06*
*relative beta band power (15-30 Hz)*	0.37	*0.10*		0.39	*0.10*	0.40	*0.09*	0.35	*0.08*		0.41	*0.08*		0.39	*0.08*
*theta/beta ratio*	0.94	*0.72*		0.63	*0.24*	0.74	*0.38*	0.92	*0.48*		0.63	*0.23*		0.74	*0.39*
**Resting Eyes Closed**															
*relative theta band power (4-7 Hz)*	0.24	*0.05*	*	0.19	*0.05*	0.22	*0.07*	0.25	*0.06*	*	0.19	*0.04*	*^t^*	0.24	*0.08*
*relative alpha band power (7-14 Hz)*	0.41	*0.05*		0.39	*0.07*	0.40	*0.07*	0.41	*0.05*		0.39	*0.08*		0.39	*0.07*
*relative beta band power (15-30 Hz)*	0.36	*0.04*	*	0.43	*0.08*	0.38	*0.08*	0.34	*0.03*	*	0.42	*0.08*	*^#^*	0.37	*0.08*
*theta/beta ratio*	0.68	*0.16*	*	0.46	*0.20*	0.62	*0.34*	0.75	*0.21*	*	0.47	*0.16*	*^t^*	0.69	*0.36*
**Continuous Performance Task**															
*relative theta band power (4-7 Hz)*	0.28	*0.06*		0.27	*0.07*	0.24	*0.07*	0.29	*0.06*		0.27	*0.06*		0.24	*0.07*
*relative alpha band power (7-14 Hz)*	0.34	*0.02*		0.34	*0.04*	0.34	*0.04*	0.35	*0.03*		0.34	*0.05*		0.33	*0.05*
*relative beta band power (15-30 Hz)*	0.37	*0.06*		0.39	*0.07*	0.41	*0.09*	0.36	*0.05*		0.39	*0.07*		0.42	*0.11*
*theta/beta ratio*	0.81	*0.28*		0.72	*0.28*	0.64	*0.25*	0.84	*0.29*		0.71	*0.25*		0.64	*0.30*

	Left Cingulate (C_3_)							Right Cingulate (C_4_)							

**Resting Eyes Open**															
*relative theta band power (4-7 Hz)*	0.27	*0.09*		0.22	*0.05*	0.24	*0.10*	0.27	*0.09*		0.22	*0.05*		0.25	*0.10*
*relative alpha band power (7-14 Hz)*	0.36	*0.02*		0.37	*0.04*	0.34	*0.06*	0.35	*0.03*		0.37	*0.04*		0.35	*0.05*
*relative beta band power (15-30 Hz)*	0.38	*0.10*		0.41	*0.05*	0.42	*0.10*	0.37	*0.09*		0.41	*0.06*		0.40	*0.09*
*theta/beta ratio*	0.87	*0.72*		0.55	*0.19*	0.69	*0.49*	0.85	*0.57*		0.57	*0.20*		0.72	*0.50*
**Resting Eyes Closed**															
*relative theta band power (4-7 Hz)*	0.23	*0.04*	*	0.19	*0.05*	0.20	*0.07*	0.24	*0.03*	*	0.19	*0.05*		0.21	*0.07*
*relative alpha band power (7-14 Hz)*	0.40	*0.04*		0.39	*0.06*	0.38	*0.06*	0.41	*0.04*		0.39	*0.06*		0.39	*0.05*
*relative beta band power (15-30 Hz)*	0.37	*0.03*	*	0.43	*0.07*	0.42	*0.09*	0.36	*0.03*	*	0.42	*0.07*		0.40	*0.07*
*theta/beta ratio*	0.64	*0.13*	*	0.46	*0.20*	0.53	*0.32*	0.66	*0.11*	*	0.47	*0.20*		0.58	*0.33*
**Continuous Performance Task**															
*relative theta band power (4-7 Hz)*	0.27	*0.04*		0.23	*0.05*	0.20	*0.05*	0.27	*0.04*		0.23	*0.05*		0.20	*0.05*
*relative alpha band power (7-14 Hz)*	0.36	*0.03*		0.35	*0.04*	0.36	*0.04*	0.36	*0.04*		0.35	*0.04*		0.36	*0.04*
*relative beta band power (15-30 Hz)*	0.37	*0.04*		0.42	*0.07*	0.45	*0.07*	0.38	*0.05*		0.42	*0.07*		0.44	*0.08*
*theta/beta ratio*	0.73	*0.19*		0.56	*0.20*	0.46	*0.17*	0.73	*0.18*		0.56	*0.17*		0.48	*0.20*

	Left Parietal (P_3_)							RightParietal (P_4_)							

**Resting Eyes Open**															
*relative theta band power (4-7 Hz)*	0.26	*0.09*		0.22	*0.05*	0.25	*0.09*	0.26	*0.10*		0.22	*0.05*		0.26	*0.10*
*relative alpha band power (7-14 Hz)*	0.39	*0.03*		0.41	*0.05*	0.39	*0.06*	0.38	*0.02*		0.41	*0.05*		0.39	*0.06*
*relative beta band power (15-30 Hz)*	0.35	*0.08*		0.38	*0.05*	0.36	*0.06*	0.36	*0.08*		0.37	*0.05*		0.35	*0.07*
*theta/beta ratio*	0.89	*0.76*		0.59	*0.19*	0.78	*0.45*	0.89	*0.81*		0.61	*0.20*		0.85	*0.60*
**Resting Eyes Closed**															
*relative theta band power (4-7 Hz)*	0.19	*0.08*		0.18	*0.05*	0.20	*0.06*	0.21	*0.04*		0.17	*0.05*		0.21	*0.06*
*relative alpha band power (7-14 Hz)*	0.46	*0.07*		0.44	*0.07*	0.45	*0.06*	0.46	*0.07*		0.44	*0.08*		0.44	*0.06*
*relative beta band power (15-30 Hz)*	0.34	*0.04*		0.38	*0.08*	0.35	*0.06*	0.34	*0.04*		0.39	*0.07*		0.35	*0.06*
*theta/beta ratio*	0.58	*0.22*		0.48	*0.21*	0.60	*0.27*	0.61	*0.10*		0.47	*0.19*		0.62	*0.30*
**Continuous Performance Task**															
*relative theta band power (4-7 Hz)*	0.26	*0.05*		0.23	*0.05*	0.21	*0.04*	0.25	*0.05*		0.23	*0.05*		0.21	*0.04*
*relative alpha band power (7-14 Hz)*	0.40	*0.04*		0.39	*0.05*	0.40	*0.05*	0.40	*0.05*		0.39	*0.05*		0.40	*0.04*
*relative beta band power (15-30 Hz)*	0.34	*0.03*		0.38	*0.06*	0.39	*0.05*	0.35	*0.03*		0.38	*0.06*		0.39	*0.05*
*theta/beta ratio*	0.77	*0.22*		0.63	*0.19*	0.55	*0.15*	0.73	*0.18*		0.64	*0.20*		0.55	*0.16*

*Significant differences between control participants and participants with bipolar disorder. ^#^Significant differences in participants with bipolarl disorder as a result of mindfulness based cognitive therapy (*p *< 0.05). *^t^*tendencies toward significant differences (*p *< 0.1). n_Control _= 10, n_Bipolar _= 12, n_Bipolar MBCT _= 12, MEAN ± STDEV.

Post MBCT individuals with bipolar disorder showed decreased beta band power over the right frontal cortex during the resting condition with eyes closed (F_4 _*t *= 2.23, diff = 0.05, Std.Dv.Diff = 0.087, *p *< 0.05) (Table 1). Theta band power (F_4 _*t *= -1.96, diff = -0.05, Std.Dv.Diff = 0.089, *p *= 0.075) and theta/beta ratio (F_4 _*t *= -2.1, diff = -0.23, Std.Dv.Diff = 0.388, *p *= 0.059) for the left frontal cortex showed a trend towards significance. No other changes in relative band power were found (Table 1).

### Brain activation (attentional task)

**Figure 1:**
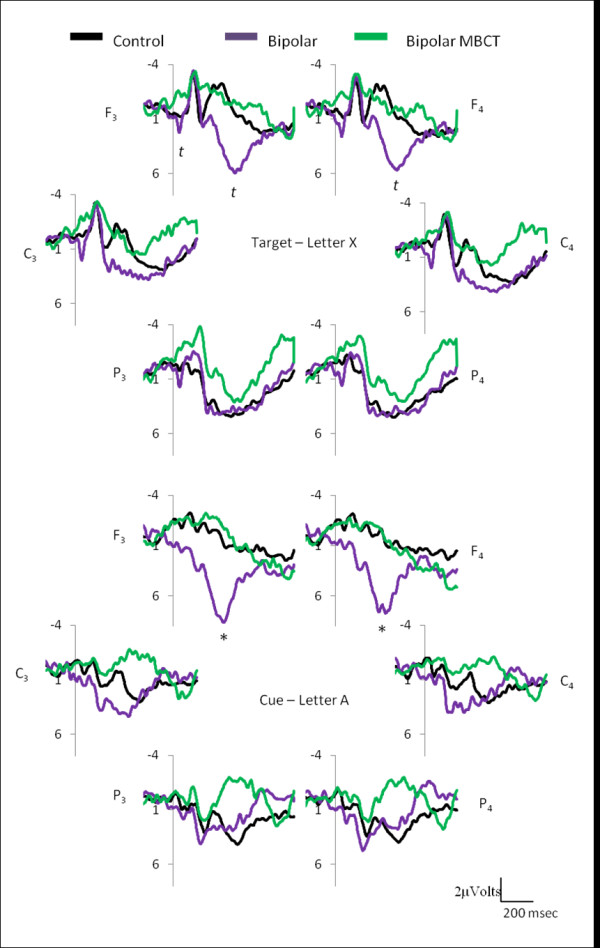
**Grand mean event-related potentials (ERPs) for frontal (F_3 _& F_4_), cingulate (C_3 _& C_4_), and parietal (P_3 _& P_4_) cortices, for the target (letter X) and the cue (letter A) of the continuous performance task**. The target ERP yielded no significant differences between controls and bipolar disorder (BD), however tendencies were found over the left frontal cortex (*t*_control vs. bipolar (0-100 ms) _= 0.07; *t*_control vs. bipolar (300-400 ms) _= 0.07) and right frontal cortex (*t*_control vs. bipolar (300-400 ms) _= 0.09). The cue yielded *significant differences between controls and bipolar disorder (BD) over the frontal cortex, as individuals with BD showed a P300-like wave component frontally and Mindfulness based cognitive therapy (MBCT) attenuated the P300-like wave component over the frontal cortices in the bipolar cohort (*p *< 0.05, *t *< 0.1, Controls_n _= 9, Bipolar_n _= 12).

Individuals with BD showed no significant differences in the target (letter X) ERP components extracted when compared with controls, although there were trends towards significance (F_3(0-100 msec) _*t *= -1.91, *p *= 0.07; F_3(300-500 msec) _*t *= -1.87, *p *= 0.07; F_4(300-500 msec) _*t *= -1.73, *p *= 0.099) (Figure 1). Individual with BD showed significant differences in the cue (letter A) ERP P300-like component when compared with controls over the frontal cortex (F_3 _*t *= -2.11; F_4 _*t *= -2.00, *p *< 0.05) (Figure 1).

Post MBCT BD individuals showed no significant differences in the target (letter X) ERP components. Post MBCT BD individuals showed a change in the P300-like waveform to one which is comparable to that seen in normal subjects during the cue (letter A) ERP over the frontal cortex (F_3 _*t *= 2.82, diff = 8.38, Std.Dv.Diff = 10.26; F_4 _*t *= 2.51, diff = 6.59, Std.Dv.Diff = 9.081, *p *< 0.05) (Figure 1).

## Discussion

Several findings emerge from this study. First, prior to MBCT brain activity in BD was greater over the frontal and cingulate cortex during resting eyes closed, which suggests decreased attentional readiness, MBCT improved attentional readiness slightly. Second, prior to MBCT brain activation in BD showed activation of non-relevant information processing over the frontal cortex, MBCT attenuated activation of this non-relevant information processing.

Brain activity prior to MBCT, individuals with BD showed decreased attentional readiness, as theta activity was decreased, beta activity was increased, and theta/beta ratios was decreased over the frontal (F3 & F4) and cingulate (C3 & C4) cortices during resting eyes closed. Increased anterior and frontal midline theta has been positively correlated with internalized attention and positive emotional state, in experienced mediators during rest [25]. During attentional processes frontal theta activity is suggested to originate from the functional connectivity between the frontal cortex and anterior cingulate cortex [26] and serves to maintain attentional processes [27], which is lacking in BD [2,13]. Furthermore increased beta activity during eyes closed has been referred to as an index of spontaneous cognitive operations of these similar brain areas, and several others areas [28]. In hand, a recent study related increased theta/beta ratios to mechanisms of approach [29]. The present data suggests that individuals with BD have deficits in resting brain activity, which may decrease their abilities to attend to relevant information, and therefore lack attentional readiness. Post MBCT intervention individuals with BD showed a slight improvement in attentional readiness, as right frontal EEG activity improved, beta activity was decreased and there was a tendency for theta and theta/beta ratios to increase. The present resting data supports the literature, that BD has 'weak' regulation of behavioral systems that are required for attentional processes [13]. MBCT intervention in BD may serve to improve attentional readiness.

Brain activation prior to MBCT individuals with BD showed activation of non-relevant information processing over the frontal cortex, as controls did not require this information processing. During the cueing process of the continuous performance task (letter A) individuals with BD showed a prominent P300-like wave form over the frontal (F3 & F4) cortices. This P300-like wave form persisted during the target but did not reach significance. Source analysis of frontal P300 wave forms has been attributed to extended cortical networks, unlike the parietal P300 wave form, at the temporal parietal junction [30]. The cueing process may assist BD by permitting activation of compensatory mechanisms, being the activation of working memory in the present attentional task [2,6,31]. Post MBCT intervention individuals with BD showed attenuated P300-like wave form over the frontal cortices. This suggests MBCT attenuated the interfering or previously required compensatory information processing brain mechanisms in BD individuals.

A number of limitations should be noted. First given the small size, we cannot determine whether some of the negative findings are false negatives, the trends towards statistical significance here may reach significance with larger sample. Second, the effect of medication on brain wave activity and/or activation were not included in the analysis, nevertheless medications were stable prior and post MBCT intervention Third, the control group did not undergo MBCT, which limits our understanding in the effects of MBCT on brain activity and activation. Fourth, no control bipolar group was included in the presented data, future studies would benefit with inclusion of a control bipolar group. Fifth, we had intended to have a gender balanced group, however during the recruitment process, mostly women volunteered, which limits the generalizability of the present study.

## Conclusions

This is the first brain imaging report on the effects of MBCT in BD. Individuals with BD show decreased attentional readiness and activated of non-relevant information processing during attentional processes. MBCT slightly improved attentional readiness, and attenuated activation of non-relevant information processing during attentional processes.

## Abbreviations

BD: Bipolar disorder; EEG: Electroencephalography; ERP: Event-related potential; MBCT: Mindfulness based cognitive therapy.

## Competing interests

The authors declare that they have no competing interests.

## Authors' contributions

FH developed the EEG protocol, undertook the EEGs, analyzed the EEG data, and drafted the manuscript. NH and ID designed the broader study of bipolar disorder within which this protocol fell, recruited and evaluated subjects, and commented on the manuscript. DS give input on the both the design of the broader bipolar protocol and the current EEG protocol, and contributed to writing of the manuscript.

## Pre-publication history

The pre-publication history for this paper can be accessed here:

http://www.biomedcentral.com/1471-244X/12/15/prepub
